# Supplementary material for: Complex‐centric proteome profiling by SEC‐SWATH‐MS
Source: Mol Syst Biol. 2019 Jan 14;15(1):e8438. doi: 10.15252/msb.20188438 (PMC6346213; doi:10.15252/msb.20188438)
Supplement: Supplementary file 7 — Dataset EV6 [file MSB-15-e8438-s007.zip › feature_plots_bioplex/P62079.pdf]

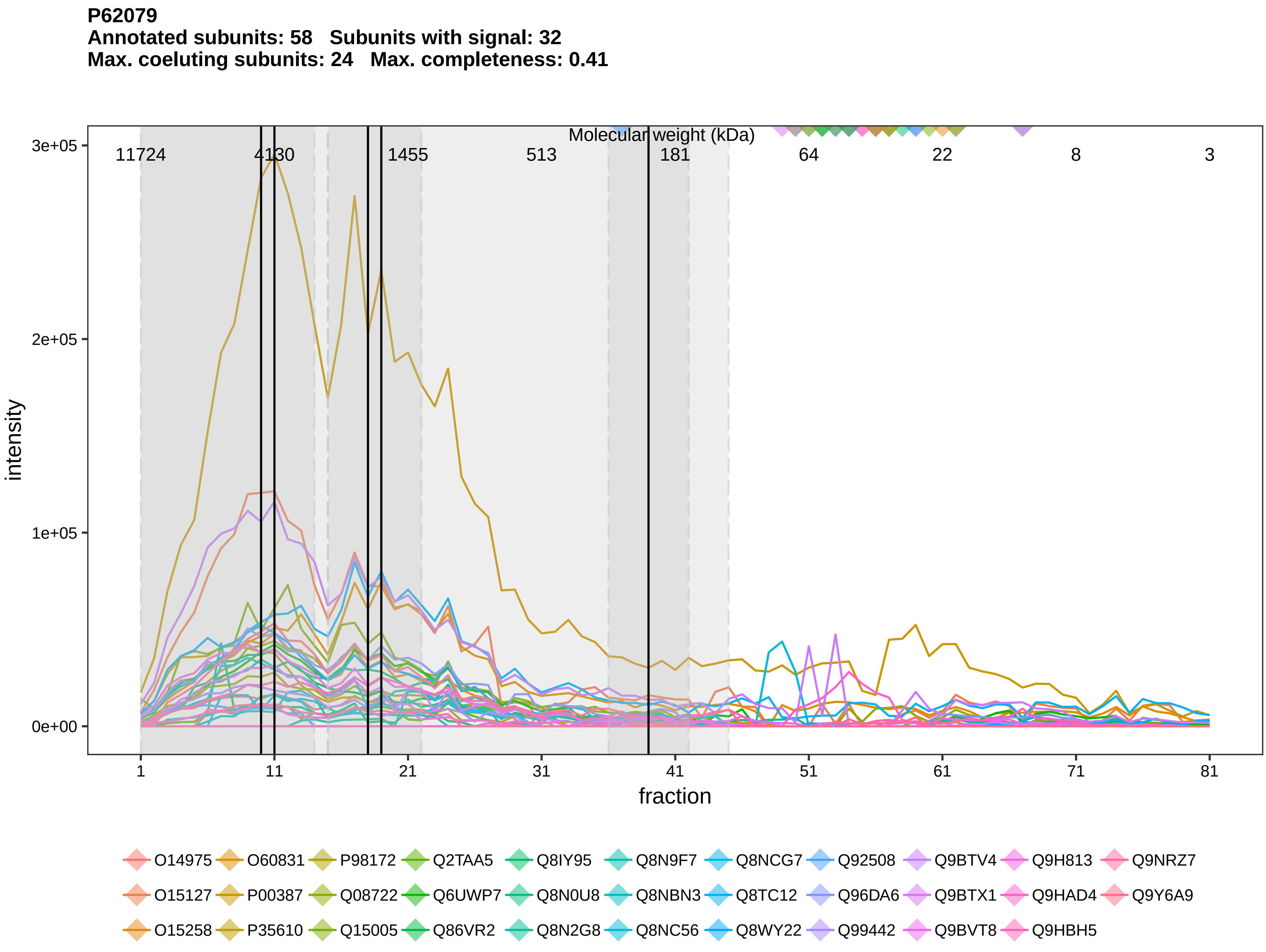
P62079
Annotated subunits: 58 Subunits with signal: 32
Max. coeluting subunits: 24 Max. completeness: 0.41
Molecular weight (kDa)
11724
4130
1455
513
181
64
22
8
3
3e+05
2e+05
1e+05
0e+00
intensity
1
11
21
31
41
51
61
71
81
fraction
O14975
O60831
P98172
Q2TAA5
Q8IY95
Q8N9F7
Q8NCG7
Q92508
Q9BTV4
Q9H813
Q9NRZ7
O15127
P00387
Q08722
Q6UWP7
Q8N0U8
Q8NBN3
Q8TC12
Q96DA6
Q9BTX1
Q9HAD4
Q9Y6A9
O15258
P35610
Q15005
Q86VR2
Q8N2G8
Q8NC56
Q8WY22
Q99442
Q9BVT8
Q9HBH5
